# 

**Published:** 1907-01

**Authors:** 


### BOOKS RECEIVED.

A Practical Treatise on Materia Medica and Therapeutics, with especial reference to the clinical application of drugs. By John V. Shoemaker, M.D., LL.D., Professor of Materia Medica, Pharmacology, Therapeutics, and Clinical Professor of Diseases of the Skin in the Medico-Chirurgical College of Philadelphia. Sixth edition. Thoroughly revised. (In conformity with latest revised U. S. Pharmacopoeia, 1905.) Octavo, 1244 pages. Philadelphia: F. A. Davis Company. (Price, Cloth, $5.00; Sheep, $6.00, net prices).

---

The Diseases of the Nose, Throat and Ear. By Charles P. Grayson, M.D., Clinical Professor of Laryngology, Medical Department, University of Pennsylvania. New (2d) edition, revised and enlarged. Octavo, about 550 pages, with 152 engravings and 15 plates in black and colors. Philadelphia and New York: Lea Brothers & Co. 1906. (Cloth, $4.00, net).

---

The Practical Medicine Series of Year Books. Ten volumes. Series of 1906. Issued under the general editorial charge of Gustavus P. Head, M. D., Professor of Laryngology and Rhinology in the Chicago Post-Graduate Medical School. Vol. VIII. Materia Medica and Therapeutics; Preventive Medicine; Climatology and Forensic Medicine. Edited by George F. Butler, Henry B. Favill, Norman Bridge, Daniel R. Brower and Harold N. Moyer. Chicago: The Year Book Publishers. (Price, $1.25; entire series, $10.00).

---

Progressive Medicine, Vol. VIII, No. 4, December, 1906. A Quarterly Digest of Advances, Discoveries and Improvements in the Medical and Surgical Sciences. Edited by Hobart Amory Hare, M.D., Professor of Therapeutics and Materia Medica in the Jefferson Medical College of Philadelphia. Octavo, 349 pages. Philadelphia and New York: Lea Brothers & Co. (Per annum, in four cloth-bound volumes, $9.00; in paper binding, $6.00; carriage paid to any address).

---

Manual of Artificial Limbs. Copiously illustrated. Octavo, pp. 430. A. A. Marks, 701 Broadway, New York.

---

Transactions of the Section of Obstetrics and Diseases of Women of the American Medical Association at the fifty-seventh annual meeting, held at Boston, June 5-8, 1906.

---

Transactions of the third and fourth annual conferences of State and Territorial Health Officers with the United States Public Health and Marine Hospital Service, held at Washington, D. C., May 15, 1905, and May 23, 1906. Two volumes. Washington: Government Printing Office.

